# First person – Claire L. Wood

**DOI:** 10.1242/dmm.043604

**Published:** 2020-01-10

## Abstract

First Person is a series of interviews with the first authors of a selection of papers published in Disease Models & Mechanisms (DMM), helping early-career researchers promote themselves alongside their papers. Claire Wood is first author on ‘A comparison of the bone and growth phenotype of *mdx*, *mdx:Cmah^−/−^* and *mdx:Utrn^+/−^* murine models with the C57BL/10 wild-type mouse’, published in DMM. Claire conducted the research described in this article while an MRC Clinical Research Training Fellow in Colin Farquharson's lab at Roslin Institute, University of Edinburgh, UK. She is now an academic clinical lecturer in the lab of Tim Cheetham and Simon Pearce at the Institute of Genetic Medicine, Newcastle University, UK, investigating bone and growth in children.


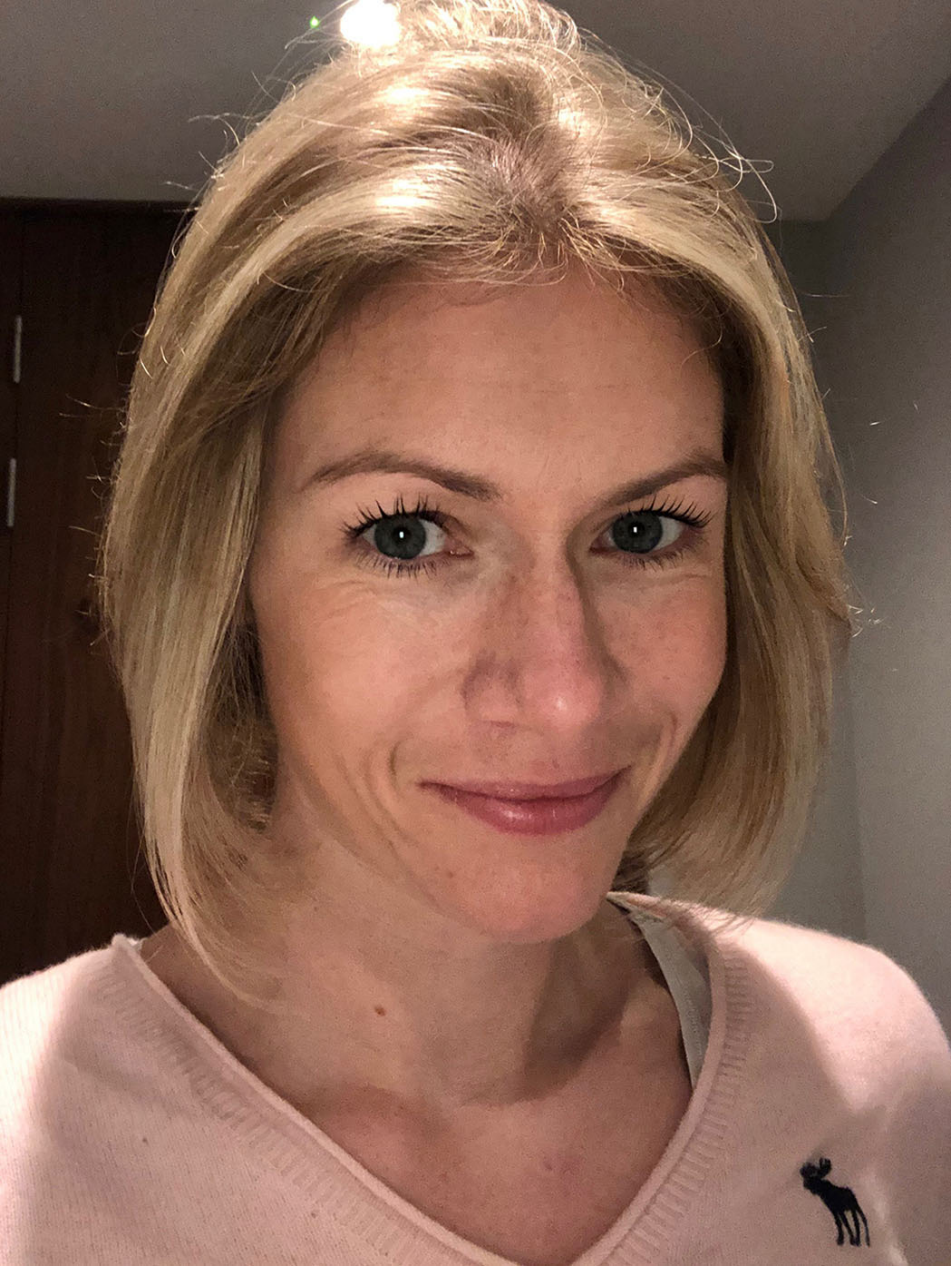


**Claire L. Wood**

**How would you explain the main findings of your paper to non-scientific family and friends?**

The mouse model commonly used in Duchenne muscular dystrophy (DMD) research is the X-linked muscular dystrophy (*mdx*) mouse, but it has significant limitations because the disease process is not as severe as in patients with DMD. Very few medications that are effective in the *mdx* mouse have shown to be useful in patients. Therefore, I have tested a new mouse model, the *mdx:Cmah^−/−^* mouse. This mouse is bred from the *mdx* mouse, but also has an additional gene deletion to make it more ‘human’ and therefore I had hoped that it would behave in a similar way to patients with DMD. I have looked, for the first time, at bone and growth in the *mdx:Cmah^−/−^* mouse and compared it to both the *mdx* and a healthy ‘wild-type’ mouse. Instead of finding impaired growth and skeletal development, I actually demonstrated that the *mdx:Cmah^−/−^* mouse shows catch-up growth and no evidence of impaired bone development.

“In order to test new agents for use in DMD, we need to continue in our quest for the most appropriate animal model to use.”

**What are the potential implications of these results for your field of research?**

Although it does not appear to be an appropriate model to investigate bone and growth in DMD, the *mdx:Cmah^−/−^* mouse may prove to be a useful model to study catch-up growth. In the meantime, in order to test new agents for use in DMD, we need to continue in our quest for the most appropriate animal model to use.

**What are the main advantages and drawbacks of the model system you have used as it relates to the disease you are investigating?**

The main advantage of the *mdx:Cmah^−/−^* mouse model that I have used is that it is genetically more similar to DMD than the traditional *mdx* mouse model and theoretically should therefore have reflected the disease course in DMD better than the *mdx* mouse. However, as I have demonstrated, the pattern of growth and bone development was not as expected.

“I am in awe of the patience that my lab colleagues have and it has surprised me how much work goes into perfecting one technique in the laboratory.”

**What has surprised you the most while conducting your research?**

This was the first time I have spent a concentrated period of time carrying out basic science research. It has been a huge learning curve for me and so different from my clinical career up until now. I am in awe of the patience that my lab colleagues have and it has surprised me how much work goes into perfecting one technique in the laboratory.

**Describe what you think is the most significant challenge impacting your research at this time and how will this be addressed over the next 10 years?**

I am currently investigating different anabolic agents to try and improve the bone and growth phenotype of steroid-treated boys with DMD. There is still no well-established small animal model that mimics DMD, and many of the compounds that have shown promise in the *mdx* mouse model have not translated into success at the clinical trial stage. This is a huge limitation when trying to re-purpose drugs.

**Figure:**
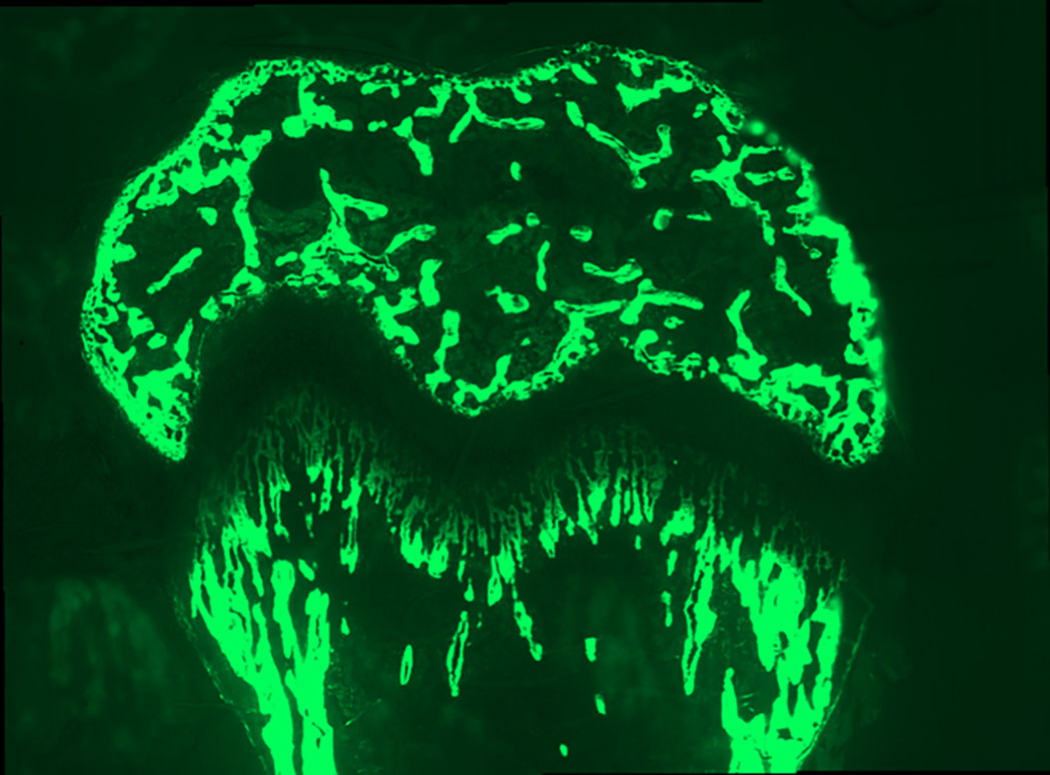
**Example of a calcein-labelled growth plate in a 3-week-old mouse.**

**What changes do you think could improve the professional lives of early-career scientists?**

I think that access to small research grants in order to continue research post-PhD and carry out pilot studies/pump-priming work prior to obtaining a larger fellowship is vital for early-career scientists, in order to be able to develop their own research interest and start to work independently.

**What's next for you?**

I have just been awarded an NIHR academic clinical lecturership at Newcastle to continue my research alongside finishing my clinical training to become a paediatric endocrinologist. I am excited to have dedicated research time to further my expertise in the field of bone and growth in paediatric endocrinology.
